# Thyroid function and hepatic fibrosis/cirrhosis: a two-sample Mendelian randomization study

**DOI:** 10.3389/fgene.2025.1399353

**Published:** 2025-04-02

**Authors:** Yan Wang, XiaoLi Zhang, Qin Li, Qing Zhang, Jun Liu

**Affiliations:** ^1^ Department of Nuclear Medicine, The Second Affiliated Hospital, JiangXi Medical College, Nanchang University, Nanchang, China; ^2^ The First Affiliated Hospital, JiangXi Medical College, Nanchang University, Nanchang, China.

**Keywords:** causal association, hepatic fibrosis/cirrhosis, hyperthyroidism, hypothyroidism, Mendelian randomization study, single-nucleotide polymorphisms, thyroid function

## Abstract

**Background:**

Evidence on the relationship between thyroid function and hepatic fibrosis/cirrhosis are still unclear, with inconsistent conclusions. This Mendelian randomization (MR) study aimed to investigate the potential causal association between thyroid function and hepatic fibrosis/cirrhosis in order to provide new insights for improving prevention and control strategies for this disease.

**Methods:**

Genome-wide association study (GWAS) data on exposures, which included hyperthyroidism, hypothyroidism, and thyroid-stimulating hormone (TSH), were extracted from the MRC Integrative Epidemiology Unit (MRC-IEU) (https://gwas.mrcieu.ac.uk/), and GWAS data for outcomes, including hepatic fibrosis/cirrhosis and chitinase-3-like protein 1 (CHI3L1), were obtained from the FinnGen consortium (https://www.finngen.fi/fi). Inverse variance weighted (IVW), weighted median, and MR-Egger methods were utilized to examine the causal association between thyroid function and the risk of hepatic fibrosis/cirrhosis. Cochran’s Q test was used to assess the heterogeneity of instrumental variables (IVs), while MR-PRESSO and leave-one-out analyses were conducted for sensitivity analysis.

**Results:**

IVW estimates suggested that hypothyroidism had a potential causal association with higher odds of hepatic fibrosis/cirrhosis (OR = 1.247, 95% CI: 1.087–1.431). Leave-one-out results indicated that this potential causal relationship was relatively robust. In addition, we assessed the causal association between hypothyroidism and hepatic fibrosis/cirrhosis before and after removal of outliers with heterogeneity. After removing the outliers, the association was still significant (OR = 1.266, 95% CI: 1.082–1.482, *P* = 0.0046).

**Conclusion:**

Patients with hypothyroidism may have a higher risk of hepatic fibrosis/cirrhosis, and this finding may provide some references for the early screening and prevention of the disease. However, further studies are needed to explore the specific mechanisms by which hypothyroidism influences hepatic fibrosis/cirrhosis.

## Introduction

Hepatic fibrosis is a significant health burden worldwide, with cirrhosis and hepatoma caused by hepatic fibrosis accounting for many hepatic disease-related deaths each year (Gines et al., 2021; Devarbhavi et al., 2023). In recent years, several observational studies have investigated the association between reduced thyroid function or the reduction in the normal range and hepatic fibrosis (Bano et al., 2020; Rahadini and Rahadina, 2022), which may be related to the regulatory role of thyroid function in lipid metabolism (Mavromati and Jornayvaz, 2021).

It has been found that subclinical hypothyroidism may be associated with hepatic fibrosis/cirrhosis (Bano et al., 2016; Kim et al., 2018); however, the conclusions are inconsistent (D'Ambrosio et al., 2021). In addition, even if thyroid function is within normal ranges, relatively high thyroid stimulating hormone (TSH) levels (Kim et al., 2019; Martinez-Escude et al., 2021a; Martinez-Escude et al., 2021b; Fan et al., 2023; Zhang et al., 2023) and low free thyroxine 4 (FT4) levels (Zhang et al., 2020; Zhang et al., 2022) are risk factors for hepatic fibrosis. However, some studies have found that there is no association between TSH (Liu et al., 2018; Zhang et al., 2020; Du et al., 2021; Guo et al., 2021) and FT4 (Du et al., 2021; Guo et al., 2021) and hepatic fibrosis. To date, few studies have discussed the relationship between hyperthyroidism and hepatic fibrosis/cirrhosis. Bano et al. (2016) found no significant association between hyperthyroidism and non-alcoholic fatty liver disease (NAFLD). In contrast, Labenz et al. (2021) suggested that hyperthyroidism is linked to a lower risk of NAFLD. In addition, serum chitinase-3-like protein 1 (CHI3L1) has recently been found to be a potential marker for the diagnosis of hepatic fibrosis and hepatic cirrhosis, but no study has investigated the relationship between thyroid function and CHI3L1 levels (Tao et al., 2014; Jin et al., 2020; Nishimura et al., 2021). The underlying mechanisms of this association remain unknown but may involve factors such as obesity, insulin resistance, oxidative stress, and mitochondrial dysfunction (Taylor et al., 2013; van Tienhoven-Wind and Dullaart, 2015; Kim et al., 2018). In conclusion, evidence on the association between thyroid function-related indices and hepatic fibrosis/cirrhosis is still limited, and observational studies cannot establish causality. Therefore, the causal association between thyroid function and hepatic fibrosis/cirrhosis needs to be further verified.

Mendelian randomization (MR) is a widely used approach to infer potential causal associations between environmental exposures and diseases (Davey Smith and Hemani, 2014; Sekula et al., 2016), which leverages single-nucleotide polymorphisms (SNPs) as instrumental variants (IVs). MR analysis can avoid reverse causality inferences and capture the long-term effects of exposures on outcomes. This study used MR methods to investigate the potential causal associations between thyroid function and hepatic fibrosis/cirrhosis, providing new insights for developing relevant screening and intervention strategies for the prevention and control of hepatic fibrosis/cirrhosis. The MR approach is widely applied in epigenetics, metabolic disease research, and environmental causal inference and has been thoroughly validated in exploring causal mechanisms. Therefore, this method can provide robust support for causal inference in the current study.

## Methods

### Data sources

In this two-sample MR study, the exposure genome-wide association study (GWAS) data, including hyperthyroidism, hypothyroidism, FT4, and TSH, were extracted from the MRC Integrative Epidemiology Unit (MRC-IEU) (https://gwas.mrcieu.ac.uk/), whereas the GWAS data for hepatic fibrosis/cirrhosis and CHI3L1 were obtained from the FinnGen consortium (https://www.finngen.fi/fi). The diagnosis of hyperthyroidism, hypothyroidism, and hepatic fibrosis/cirrhosis was according to the International Classification of Diseases (ICD) 10th Version, with codes of E05, E03, and K74, respectively. We summarized and presented the aggregated information on the data source, GWAS ID, population, and sample size in Table 1.

**TABLE 1:** Aggregated data of study exposures and outcomes.

GWAS ID	Trait	ICD-10	Total sample (case)	PMID/Consortium
Exposure				
ebi-a-GCST90018860	Hyperthyroidism	E05	460,499 (3,557)	34594039
ebi-a-GCST90018862	Hypothyroidism	E03	410,141 (30,155)	34594039
prot-c-3521_16_2	TSH		997	28240269
GCST90012662	FT4		26,231	33441150
Outcomes				
finn-b-K11_FIBROCHIRLIV	Hepatic fibrosis/cirrhosis	K74	214,403 (811)	FinnGen
ebi-a-GCST90010201	CHI3L1 levels		1,323	33303764

ICD-10, the International Classification of Diseases (ICD) 10th Version; TSH, thyroid stimulating hormone; FT4, free thyroxine; CHI3L1, chitinase-3-like protein 1.

The GWAS datasets in this study received ethical approval from their respective institutions, with data de-identified, and informed consent was obtained from all participants. Since the database is publicly available, ethical approval was waived by the Institutional Review Board (IRB) of our hospital. In addition, the methods and procedures of this study followed the STROBE-MR checklist to ensure the robustness and reliability of the findings (Skrivankova et al., 2021).

### Selection of single-nucleotide polymorphisms

SNPs significantly associated with exposures were selected as potential IVs, with a selection threshold of *P* < 5.0 × 10^−8^ (*P* < 5.0 × 10^−6^ for IVs associated with FT4 and TSH). SNPs in linkage disequilibrium (LD) or those that were palindromic with intermediate allele frequencies were removed according to the MR principle to ensure that the same allele corresponded to the effects between SNPs and both the exposures and outcomes. The threshold was set to be r^2^ = 0.001, with a clumping distance of 10,000 kb.

### Assumptions of the MR analysis

The MR analysis must adhere to three important assumptions in order to minimize bias in the results. (1) Relevance assumption: the selected IVs must exhibit significant associations with the exposure factor (P < 5.0 × 10^−8^ or *P* < 5.0 × 10^−6^). (2) Independence assumption: IVs should be independent of potential confounders. To validate this, we implemented LD clumping procedures for IV selection. Details regarding assumptions (1) and (2) can be found in the section on the selection of single-nucleotide polymorphisms. Horizontal pleiotropy was systematically evaluated using the MR-PRESSO global test (P < 0.05 as the significance threshold) and MR-Egger regression. (3) Exclusion restriction assumption: IVs should influence outcomes solely through exposure. The PhenoScanner V2 database was used to exclude SNPs with documented pleiotropic pathways. Additionally, the intercept term of the MR-Egger regression was examined to assess residual horizontal pleiotropy (A P-value > 0.05 was considered to indicate the absence of horizontal pleiotropic effects).

First, IVs need to be significantly associated with exposures. The strength of association between exposures and IVs was estimated using the following formula: F = r^2^ * (N-2)/(1-r^2^), where r^2^ = 2 * EAF * (1-EAF) *β^2^/SD^2^. Here, N is the sample size, EAF is the effect allele frequency, β represents the regression coefficient for the association between exposures and IVs, and SD is the standard deviation. In addition, F < 10 indicates a weak association between IVs and exposures. Second, IVs must be independent of confounders associated with exposures and outcomes. To determine whether this assumption was violated, the MR-Egger regression test was utilized to detect potential horizontal pleiotropy, which refers to confounding effects caused by other diseases (Burgess and Thompson, 2017; Bowden et al., 2019). A significant intercept term in MR-Egger represents the existence of pleiotropy. Third, IVs should affect outcomes solely through exposure, meaning that there should be no horizontal pleiotropy influence of IVs on the outcome.

### Statistical analysis

The potential causal association between thyroid function and hepatic fibrosis/cirrhosis was estimated using the inverse variance weighted (IVW) method, which was evaluated by odds ratios (ORs) with 95% confidence intervals (CIs). The IVW test serves as the primary method to calculate the unbiased estimates of causal effect when horizontal pleiotropy is absent. The weighted median method was also used to assess the causal effect because it can provide a robust and consistent estimate even if nearly 50% of the genetic variants are invalid instruments. The MR-Egger regression was used as its intercept allows for the detection of potential pleiotropy in IVs (*P* > 0.05 was recognized as no horizontal pleiotropy). In addition, false discovery rate (FDR) correction was utilized to increase the reliability and stability of the results. A *P* < 0.05 value was considered statistically significant for the potential causal relationships.

The heterogeneity of the significant causal effect was tested using Cochran’s Q test, where IVs with *P* < 0.05 were considered heterogeneous. Outliers among selected IVs were identified using the MR-Pleiotropy RESidual Sum and Outlier (MR-PRESSO) test (Verbanck et al., 2018). The causal associations between thyroid function and hepatic fibrosis/cirrhosis were assessed after excluding significant outliers. The scatter plots and the leave-one-out test were used for sensitivity analysis. Statistical analyses were performed with R version 4.2.0 (Institute for Statistics and Mathematics, Vienna, Austria). The R package “TwoSampleMR” was used for MR analyses of the causal association between thyroid function and hepatic fibrosis/cirrhosis.

## Results

**FIGURE 1:**
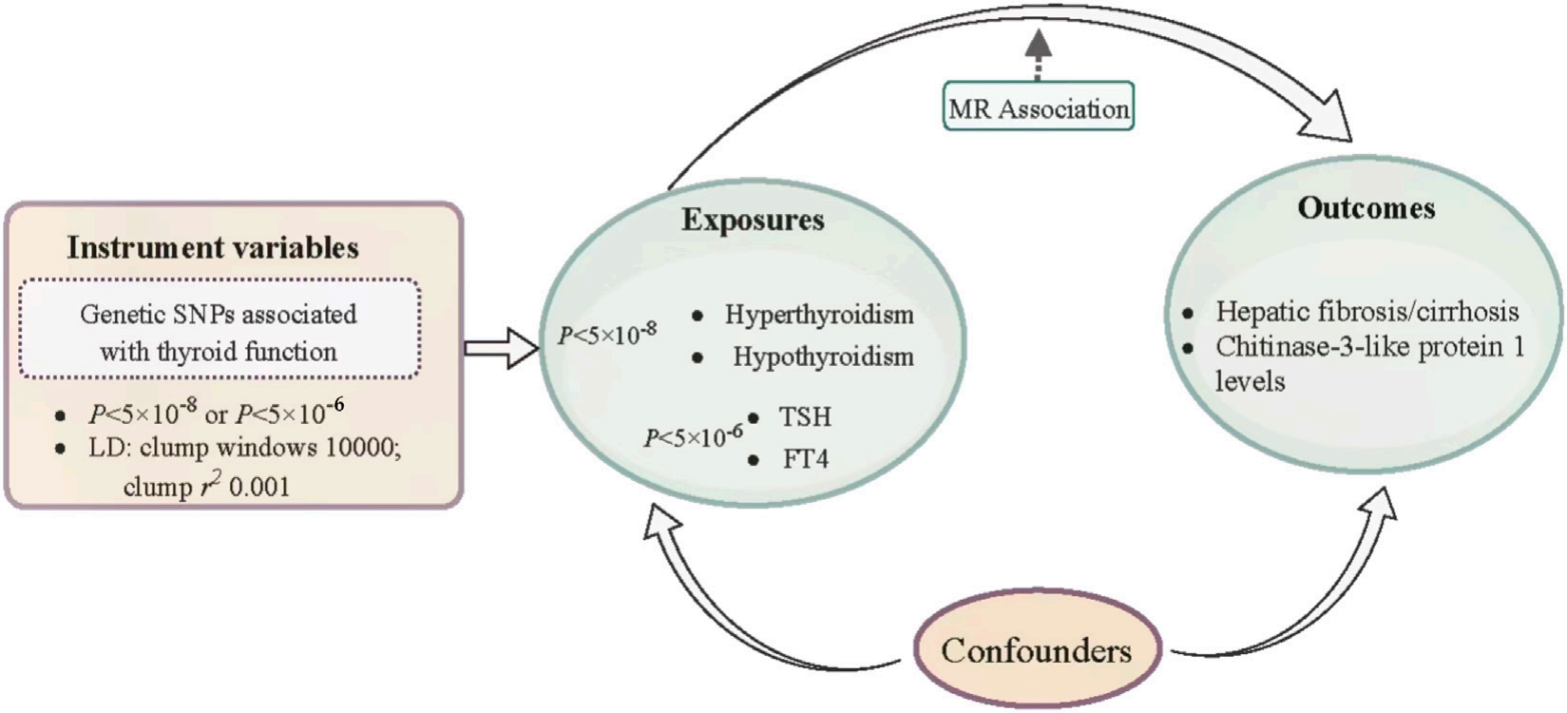
Flowchart of the study process.

The flowchart of the study process is shown in Figure 1. Table 2 shows the selection of IVs and the results of tests for horizontal pleiotropy, strength, and heterogeneity. After quality control, we identified 11 SNPs (related to hyperthyroidism), 62 SNPs (related to hypothyroidism), and 4 SNPs (related to TSH) as IVs for the exposures. The MR-Egger regression test indicated no evidence of horizontal pleiotropy (all *P* > 0.05). The strength values of the associations between IV exposures were all greater than 20. However, Cochran’s Q test showed potential heterogeneity in the associations between selected IVs and hyperthyroidism (*P =* 1.09 × 10^−4^) and hypothyroidism (*P* = 0.006).

**TABLE 2:** IV selection and testing for horizontal pleiotropy and strength.

Outcome	Exposure	Selected SNPs (*P* < 5 × 10^−8^ or *P* < 5 × 10^−6^)	Omitted LD SNPs	Drop all palindromic SNPs	Strength		Horizontal pleiotropy test		Heterogeneity			
					F	R^2^ (%)	MR-Egger intercept	*P*	MR Egger Q	*P*	IVW	*P*
Hepatic fibrosis/cirrhosis	Hyperthyroidism	8,296	12	11	65.127	0.002	0.02	0.823	33.69	**2.08 × 10** ^**−4**^	33.49	**1.09 × 10** ^**−4**^
	Hypothyroidism	20,340	69	62	78.659	0.013	−0.01	0.523	92.01	**0.006**	91.38	**0.006**
	FT4	60	11	11	29.132	0.012	0.03	0.519	5.06	0.887	4.61	0.867
	TSH	7	5	4	22.093	0.109	−0.11	0.550	2.37	0.499	1.86	0.394
CHI3L1	Hyperthyroidism	8,296	12	11	65.127	0.002	−0.06	0.823	10.80	0.374	8.21	0.513
	Hypothyroidism	20,340	69	62	81.143	0.013	−0.01	0.484	58.67	0.561	58.18	0.543
	FT4	60	11	11	29.132	0.012	−0.02	0.729	15.15	0.127	14.94	0.093
	TSH	7	5	4	22.092	0.109	0.24	0.221	5.47	0.140	2.15	0.342

IV, instrumental variable; SNP, single-nucleotide polymorphism; LD, linkage disequilibrium; MR, Mendelian randomization; F = r^2^ * (N-2)/(1-r^2^), where r^2^ = 2 * EAF * (1-EAF) *β^2^/SD^2^.

IVW, inverse variance weighted; FT4, free thyroxine; TSH, thyroid stimulating hormone. P < 0.05 as the significance threshold.

**FIGURE 2:**
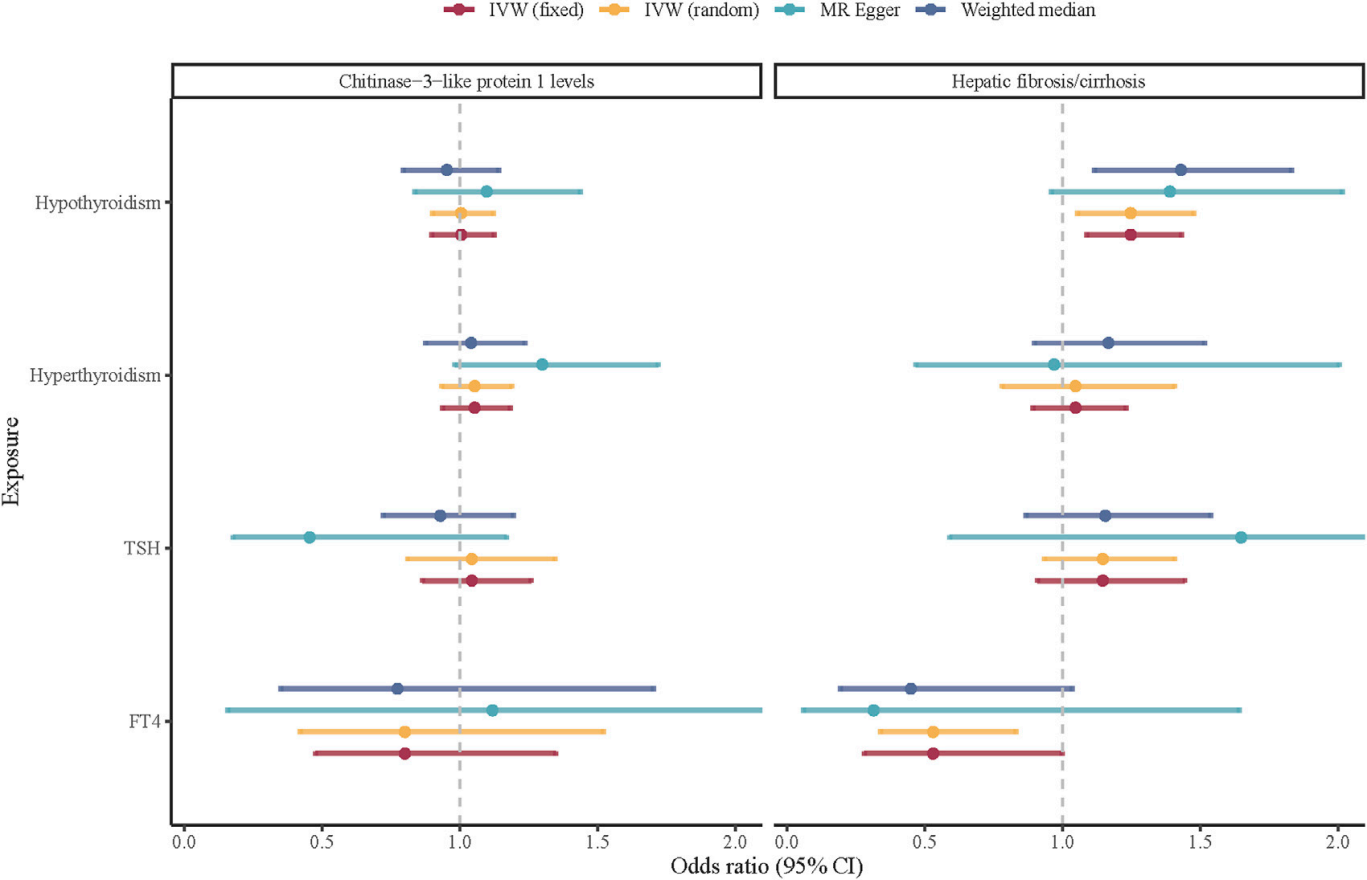
Causal associations between thyroid function and hepatic fibrosis/cirrhosis.

Then, we investigated the potential causal associations between thyroid function and hepatic fibrosis/cirrhosis (Figure 2). After applying the FDR correction, the IVW estimate suggested that hypothyroidism had a positive causal association with hepatic fibrosis/cirrhosis (OR = 1.247, 95% CI: 1.087–1.431), while FT4 was negatively associated with the odds of hepatic fibrosis/cirrhosis (OR = 0.530, 95% CI: 0.282–0.998). In addition, weighted median results similarly suggested a positive causal association between hypothyroidism and hepatic fibrosis/cirrhosis (OR = 1.430, 95% CI: 1.123–1.820). More detailed information on the causal associations between thyroid function and hepatic fibrosis/cirrhosis is provided in Supplementary Table S1.

**FIGURE 3:**
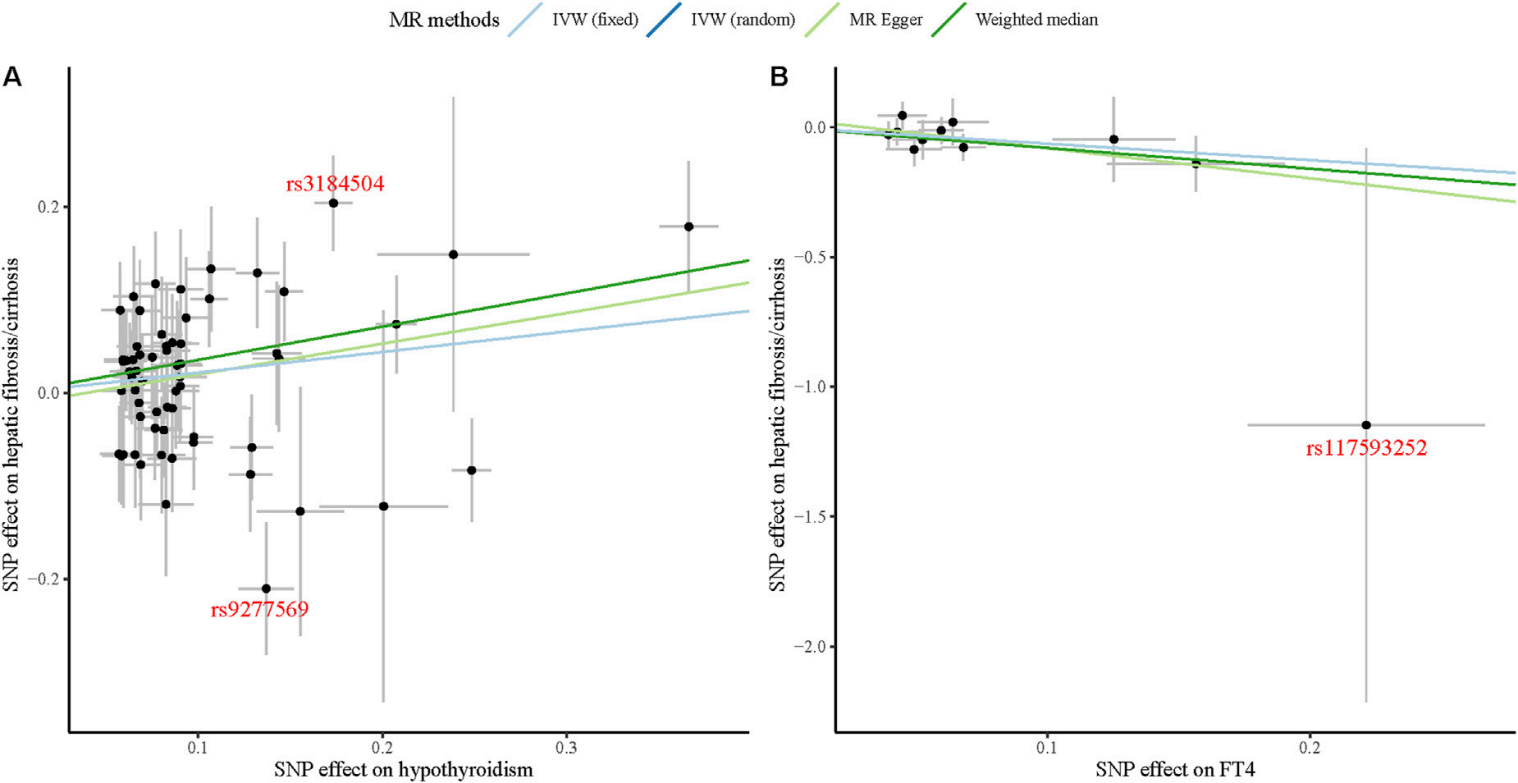
Scatter plots of causal associations between both hypothyroidism **(A)** and FT4 **(B)** and hepatic fibrosis/cirrhosis.

**FIGURE 4:**
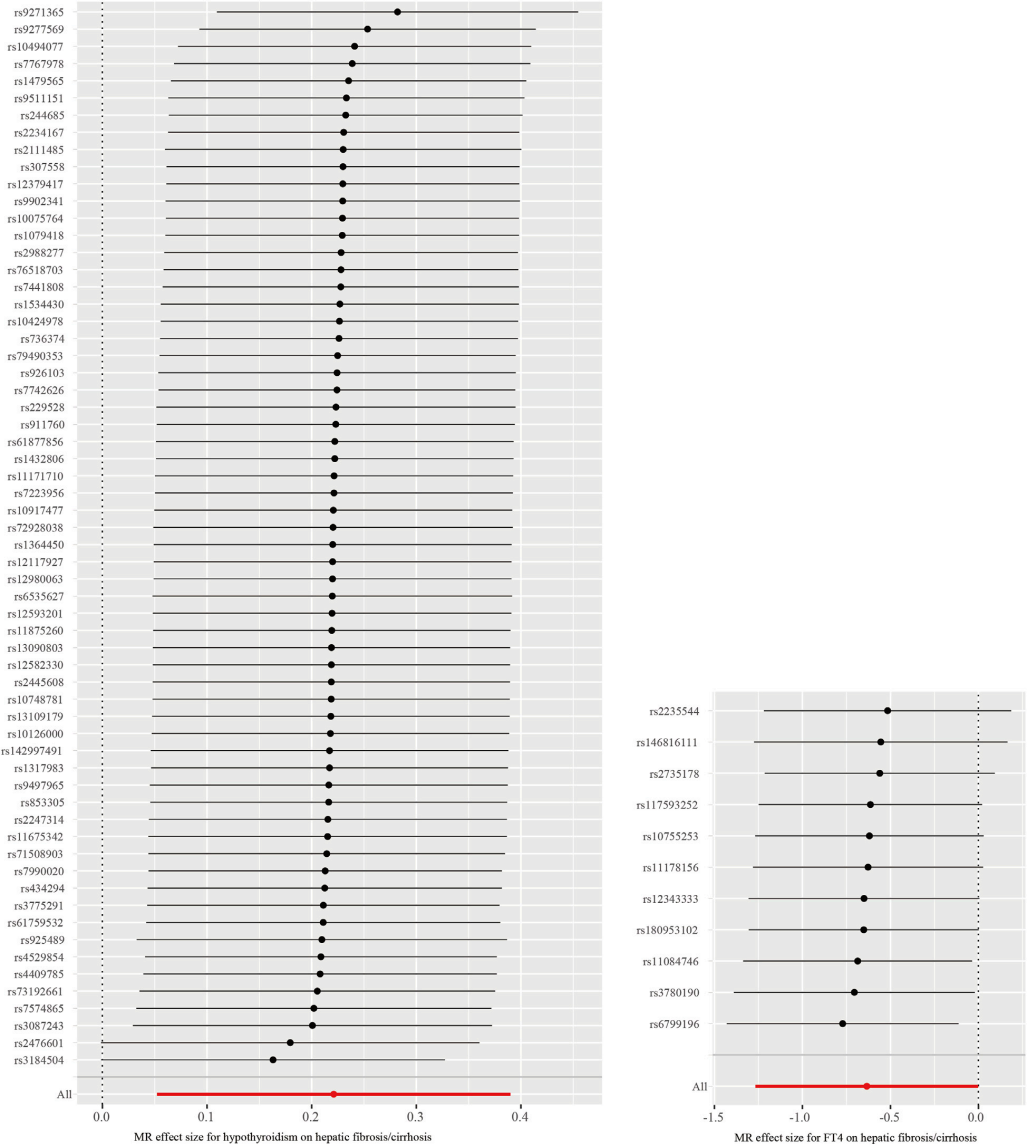
Leave-one-out test of causal associations between both hypothyroidism and FT4 and hepatic fibrosis/cirrhosis.

Through the MR-PRESSO global test, we identified two significant outliers among the IVs associated with hypothyroidism and hepatic fibrosis/cirrhosis and one for the association between FT4 and hepatic fibrosis/cirrhosis. It could also be clearly observed in Figure 3 that the tendency of causal associations between hypothyroidism and FT4 and hepatic fibrosis/cirrhosis evaluated using different MR methods was substantially consistent. Then, we confirmed the reliability of the MR results regarding the potential causal associations between both hypothyroidism and FT4 and hepatic fibrosis/cirrhosis through sensitivity analysis. The leave-one-out test indicated that the potential causal effects of hypothyroidism and FT4 on the risk of hepatic fibrosis/cirrhosis were relatively robust (Figure 4).

Moreover, since the heterogeneity test indicated that there was heterogeneity in the associations between selected IVs and hypothyroidism, we assessed the causal relationship between hypothyroidism and hepatic fibrosis/cirrhosis before and after removal of significant outliers (Table 3). According to the IVW results, after the removal of outliers with heterogeneity, the causal association between hypothyroidism and hepatic fibrosis/cirrhosis was still significant (OR = 1.217, 95% CI: 1.055–1.404) (Table 4).

**TABLE 3:** Causal relationship between thyroid function and hepatic fibrosis/cirrhosis before and after removal of outliers with heterogeneity.

Outcome	Exposure	SNPs	MR analysis	Global test	OR (95% CI)	*P*
Hepatic fibrosis/cirrhosis	Hyperthyroidism	11	Raw data	<0.001	1.0589 (0.8031–1.3961)	0.6927
	Hyperthyroidism	10	Outlier-eliminated		1.1286 (0.8881–1.4341)	0.3459
	Hypothyroidism	62	Raw data	0.004	1.2279 (1.0415–1.4476)	**0.0171**
	Hypothyroidism	60	Outlier-eliminated		1.2661 (1.0815–1.4822)	**0.0046**

MR, Mendelian randomization; OR, odds ratio; CI, confidence interval. P < 0.05 as the significance threshold.

**TABLE 4:** Causal association between thyroid function and hepatic fibrosis/cirrhosis after removal of outliers.

Outcome	Exposure	Method	SNP	OR (95% CI)	*P*
Hepatic fibrosis/cirrhosis	FT4	IVW (fixed)	10	0.541 (0.287–1.020)	0.058
		IVW (random)	10	0.541 (0.352–0.833)	**0.005**
		MR Egger	10	0.346 (0.065–1.835)	0.248
		Weighted median	10	0.451 (0.198–1.025)	0.057
	Hyperthyroidism	IVW (fixed)	10	1.119 (0.949–1.320)	0.183
		IVW (random)	10	1.119 (0.865–1.447)	0.393
		MR Egger	10	0.935 (0.508–1.718)	0.833
		Weighted median	10	1.230 (0.952–1.587)	0.113
	Hypothyroidism	IVW (fixed)	60	1.217 (1.055–1.404)	**0.007**
		IVW (random)	60	1.217 (1.041–1.422)	**0.013**
		MR Egger	60	1.291 (0.917–1.818)	0.148
		Weighted median	60	1.425 (1.115–1.822)	**0.005**

SNP, single-nucleotide polymorphism; OR, odds ratio; CI, confidence interval; IVW, inverse variance weighted; MR, Mendelian randomization. P < 0.05 as the significance threshold.

## Discussion

We conducted a two-sample MR analysis to explore the causal associations between different thyroid function-related indices and hepatic fibrosis/cirrhosis. The results showed that hypothyroidism had a positive causal relationship with hepatic fibrosis/cirrhosis, whereas FT4 had a negative association.

In recent years, the relationship between thyroid function and hepatic fibrosis/cirrhosis has been extensively studied. However, existing evidence from animal experiments or observational studies cannot infer a causal relationship between them. Research in patients from Jiading District Central Hospital, Shanghai, China, showed that low-normal thyroid function increased the risk of advanced fibrosis in patients with metabolic dysfunction-associated fatty liver disease (MAFLD), and the elevated TSH concentrations were linked to advanced hepatic fibrosis (Fan et al., 2023). A cross-sectional study in the Indian population suggested that patients with hepatic cirrhosis showed impaired thyroid function, and that thyroid hormone (TH) levels may help assess the severity and course of cirrhosis (Sikarwar et al., 2022). In a mouse model of non-alcoholic steatohepatitis, TH was observed to reduce lipotoxicity, hepatic inflammation, oxidative stress, and fibrosis (Zhou et al., 2022). An MR study suggested an inverse association between genetically predicted hypothyroidism and hepatocellular carcinoma risk, but there was no evidence observed of a direct causal effect of TSH level and FT4 level on HCC (Lu et al., 2022). On the other hand, Shao et al. (2022) found that FT4 in the normal range was lower in the NAFLD group than in the healthy control group. In the current MR study, we found that patients with hypothyroidism had a higher hepatic fibrosis/cirrhosis risk, while FT4 was negatively associated with the odds of hepatic fibrosis/cirrhosis; however, no significant causal association was observed between TSH and hepatic fibrosis/cirrhosis. Our findings were relatively complementary to the existing literature by filling the gap in inferring causal associations between thyroid function and both hepatic fibrosis and cirrhosis. Furthermore, the observed discrepancies between our findings and prior studies may be attributable to population stratification by ancestry. These preliminary causal associations therefore warrant validation through large-scale multi-ethnic cohorts to elucidate potential ancestry-specific effects.

The mechanisms through which hypothyroidism and FT4 may influence the occurrence of hepatic fibrosis/cirrhosis are still unclear. The liver plays an essential physiological role in TH activation and inactivation, transport, and metabolism, and conversely, TH affects the activities of hepatocytes and hepatic metabolism (Piantanida et al., 2020). Kannt et al. (2021) showed that treatment with hepatic thyroid hormone receptor β (THR-β) in patients with non-alcoholic steatohepatitis (NASH) led to a significant reduction in hepatic steatosis, α-smooth muscle actin content, and the expression of genes involved in fibrogenesis, indicating a decrease in hepatic fibrosis. An upregulation of THR-β was observed in the developing brain under iodine deficiency, indicating an adaptive process coming into play to protect it from the damages that are inflicted due to hypothyroidism (Chattopadhyay et al., 1995). Hence, it is suggested that hypothyroidism may be involved in the development of hepatic fibrosis/cirrhosis by influencing the relative expression of THR-β. In addition, hypothyroidism in hepatic cirrhosis inevitably affects mitochondrial metabolism and functional integrity, as the liver is one of the main target organs for THs (Weitzel et al., 2003). It is known that hypothyroidism in hepatic cirrhosis influences mitochondrial oxidative phosphorylation and disrupts the function of many antioxidant enzymes (Mukherjee et al., 2014). The excessive accumulation of free oxygen radicals in hepatocytes may play a critical role in the development of hepatocellular carcinoma (HCC) in cirrhosis by causing oxygen-free radical-related DNA damage (Sahin et al., 2020). Nevertheless, due to the limitation of GWAS databases, other indices related to thyroid function, such as TH, THR-β, triiodothyronine (T3), and thyroxine (T4), were not available. Therefore, the specific underlying mechanisms behind the causal associations between both hypothyroidism and FT4 and the occurrence of hepatic fibrosis/cirrhosis need to be further explored and elucidated.

As mentioned above, the MR study design is superior to observational studies in clarifying the causal effect of potential risk factors for diseases of interest to a certain extent. By investigating the potential causal association that hypothyroidism increases the odds of hepatic fibrosis/cirrhosis and FT4 decreases the odds of hepatic fibrosis/cirrhosis, our study may provide some references for further exploration to facilitate the recommendation of public health policies and clinical interventions among patients with hepatic diseases and help reduce the incidence and social burden of this disease. Compared with previous research, the current study included a large sample of the European population from the open-access GWAS databases. According to our findings, clinicians should focus on the regular screening and timely treatment of hepatic inflammation or fibrosis in patients with hypothyroidism and monitor serum FT4 levels to maintain them within an appropriate range in order to reduce the further risk of developing hepatic cirrhosis.

The MR method can overcome the limitations of some observational studies such as reverse causality, confounding factors, and various biases. In the current research, the selected IVs for the MR analysis were screened rigorously, with F values of greater than 20, guaranteeing the accuracy of the results. Although the heterogeneity test showed that the selected IVs related to hyperthyroidism and hypothyroidism had potential heterogeneity, we utilized the MR-PRESSO distortion test to screen significant outliers and assessed the causal association between hypothyroidism and hepatic fibrosis/cirrhosis before and after deleting outliers (Verbanck et al., 2018). The leave-one-out method was used for sensitivity analysis. All these tests suggested that the causal associations between both hypothyroidism and FT4 and hepatic fibrosis/cirrhosis were stable and robust. However, there are some limitations. This study included only European populations, so the findings may not be generalizable for extrapolation to individuals of other racial or ethnic backgrounds. Additionally, the GWAS data we used were aggregated rather than individual level, preventing stratification by age, gender, and other factors for further analysis. We also fully acknowledge the critical role of population stratification effects in two-sample MR studies. To address this concern, all GWAS datasets utilized in our analysis incorporated principal component analysis (PCA)-based ancestry adjustment in their original studies, which serves as a methodological safeguard against confounding by population structure. Furthermore, we recognize the importance of expanding population diversity in future research. We propose to validate these associations through large-scale, multi-ancestry analyses to ensure result robustness (Genevieve et al., 2019).

## Conclusion

Hypothyroidism may be a potential risk factor for hepatic fibrosis/cirrhosis, while FT4 exhibits a negative causal association with these conditions, indicating that dynamic monitoring and screening of relevant indices may be significant for individuals at high risk of hepatic fibrosis and cirrhosis. Further studies are needed to elucidate the underlying mechanisms of these associations.

## Data Availability

The original contributions presented in the study are included in the article/Supplementary Material; further inquiries can be directed to the corresponding author.

## Abbreviations

TSH, thyroid stimulating hormone; FT4, free thyroxine 4; NAFLD, non-alcoholic fatty liver disease; CHI3L1, chitinase-3-like protein 1; MR, Mendelian randomization; SNP, single-nucleotide polymorphism; IV, instrumental variant; GWAS, genome-wide association study; MRC-IEU, MRC Integrative Epidemiology Unit; ICD, International Classification of Diseases; IRB, Institutional Review Board; LD, linkage disequilibrium; IVW, inverse variance weighted; OR, odds ratio; CI, confidence interval; FDR, false discovery rate; MR-PRESSO, MR-Pleiotropy RESidual Sum and Outlier; MAFLD, metabolic dysfunction-associated fatty liver disease; TH, thyroid hormone; THR-β, thyroid hormone receptor β; NASH, non-alcoholic steatohepatitis; HCC, hepatocellular carcinoma; T3, triiodothyronine; T4, thyroxine.
