# Supplementary material for: Thyroid function and hepatic fibrosis/cirrhosis: a two-sample Mendelian randomization study
Source: Front Genet. 2025 Apr 2;16:1399353. doi: 10.3389/fgene.2025.1399353 (PMC11999943; doi:10.3389/fgene.2025.1399353)
Supplement: Supplementary file 1 [file Table1.DOCX]

Table S1 Potential causal association of thyroid function with hepatic fibrosis/cirrhosis

| Outcomes | Exposures | Methods | OR (95% CI) | *P* | *P_FDR_* |
| --- | --- | --- | --- | --- | --- |
| Hepatic fibrosis/cirrhosis | Hyperthyroidism | IVW (fixed) | 1.047 (0.892-1.230) | 0.575 | 0.657 |
|  |  | IVW (random) | 1.047 (0.780-1.406) | 0.760 | 0.811 |
|  |  | MR Egger | 0.969 (0.468-2.005) | 0.934 | 0.934 |
|  |  | Weighted median | 1.166 (0.903-1.507) | 0.238 | 0.362 |
|  | Hypothyroidism | IVW (fixed) | 1.247 (1.087-1.431) | **0.002** | **0.026** |
|  |  | IVW (random) | 1.247 (1.054-1.477) | **0.010** | **0.041** |
|  |  | MR Egger | 1.390 (0.959-2.015) | 0.087 | 0.199 |
|  |  | Weighted median | 1.430 (1.123-1.820) | **0.004** | **0.030** |
|  | FT4 | IVW (fixed) | 0.530 (0.282-0.998) | **0.049** | 0.158 |
|  |  | IVW (random) | 0.530 (0.338-0.832) | **0.006** | **0.030** |
|  |  | MR Egger | 0.314 (0.060-1.641) | 0.203 | 0.361 |
|  |  | Weighted median | 0.450 (0.190-1.064) | 0.069 | 0.161 |
|  | TSH | IVW (fixed) | 1.146 (0.910-1.444) | 0.247 | 0.362 |
|  |  | IVW (random) | 1.146 (0.933-1.408) | 0.193 | 0.361 |
|  |  | MR Egger | 1.648 (0.591-4.591) | 0.440 | 0.542 |
|  |  | Weighted median | 1.155 (0.867-1.538) | 0.324 | 0.432 |
| CHI3L1 levels | Hyperthyroidism | IVW (fixed) | 1.054 (0.937-1.184) | 0.381 | 0.878 |
|  |  | IVW (random) | 1.054 (0.933-1.189) | 0.399 | 0.878 |
|  |  | MR Egger | 1.299 (0.981-1.719) | 0.101 | 0.878 |
|  |  | Weighted median | 1.041 (0.880-1.231) | 0.643 | 0.878 |
|  | Hypothyroidism | IVW (fixed) | 1.004 (0.898-1.124) | 0.941 | 0.941 |
|  |  | IVW (random) | 1.004 (0.900-1.121) | 0.940 | 0.941 |
|  |  | MR Egger | 1.097 (0.837-1.438) | 0.504 | 0.878 |
|  |  | Weighted median | 0.953 (0.796-1.140) | 0.597 | 0.878 |
|  | TSH | IVW (fixed) | 0.800 (0.475-1.349) | 0.403 | 0.878 |
|  |  | IVW (random) | 0.800 (0.421-1.521) | 0.497 | 0.878 |
|  |  | MR Egger | 1.118 (0.159-7.887) | 0.913 | 0.941 |
|  |  | Weighted median | 0.774 (0.367-1.629) | 0.499 | 0.878 |

OR: odds ratio, CI: confidence interval, MR: Mendelian randomization, IVW: inverse variance weighted, TSH: thyroid stimulating hormone.
