# Supplementary material for: Thyroid function and hepatic fibrosis/cirrhosis: a two-sample Mendelian randomization study
Source: Front Genet. 2025 Apr 2;16:1399353. doi: 10.3389/fgene.2025.1399353 (PMC11999943; doi:10.3389/fgene.2025.1399353)
Supplement: Supplementary file 2 [file DataSheet1.ZIP › Raw Data 原始数据和代码-甲状腺功能与肝纤维化肝硬化/Figuers and Tables/Tables.docx]

Table 1. Aggregated data of study exposures and outcomes.

| GWAS ID | Trait | Year | Sample size | Population | PMID/ Consortium |
| --- | --- | --- | --- | --- | --- |
| **Exposures** |  |  |  |  |  |
| ebi-a-GCST90018860 | Hyperthyroidism | 2021 | 460,499 | European | 34594039 |
| ebi-a-GCST90018862 | Hypothyroidism | 2021 | 410,141 | European | 34594039 |
| prot-c-3521_16_2 | TSH | 2019 |  | European | 28240269 |
| **Outcomes** |  |  |  |  |  |
| finn-b-K11_FIBROCHIRLIV | Hepatic fibrosis/cirrhosis | 2021 | 214,403 | European | Finngen  (https://www.finngen.fi/fi.) |
| ebi-a-GCST90010201 | CHI3L1 levels | 2020 | 1,323 | European | 33303764 |

TSH: thyroid stimulating hormone, CHI3L1: chitinase-3-like protein 1.

Table 2 IVs selection and test for horizontal pleiotropy and strength.

| Outcomes | Exposures | Selected SNPs  (*P*<5×10^-8^) | Omitted LD SNPs | Drop all palindromic SNPs | Strength | | Horizontal pleiotropy test | | Heterogeneity | | | |
| --- | --- | --- | --- | --- | --- | --- | --- | --- | --- | --- | --- | --- |
|  |  |  |  |  | F | R^2^ (%) | MR Egger intercept | *P* | MR Egger Q | *P* | IVW | *P* |
| Hepatic fibrosis/cirrhosis | Hyperthyroidism | 8296 | 12 | 11 | 65.013 | 0.014 | 0.0197 | 0.8226 | **33.4950** | **0.0001** | **33.6932** | **0.0002** |
|  | Hypothyroidism | 20340 | 69 | 62 | 78.623 | 0.019 | -0.0127 | 0.5228 | **91.3789** | **0.0056** | **92.0083** | **0.0063** |
|  | TSH | 7 | 5 | 4 | 22.093 | 2.176 | -0.1071 | 0.5499 | 1.8635 | 0.3939 | 2.3716 | 0.4989 |
| CHI3L1 | Hyperthyroidism | 8296 | 12 | 11 | 65.013 | 0.014 | -0.0569 | 0.1424 | 8.2127 | 0.5129 | 10.7963 | 0.3736 |
|  | Hypothyroidism | 20340 | 69 | 62 | 81.097 | 0.020 | -0.0099 | 0.4841 | 58.1760 | 0.5427 | 58.6719 | 0.5608 |
|  | TSH | 7 | 5 | 4 | 22.093 | 2.176 | 0.2431 | 0.2205 | 2.1482 | 0.3416 | 5.4745 | 0.1402 |

IV: instrumental variables, SNP: single nucleotide polymorphism, LD: linkage disequilibrium, MR: Mendelian randomization, F = r^2^ * (N-2) / (1-r^2^), r^2^ = 2 * EAF * (1-EAF) * β^2^ / SD^2^, IVW: inverse variance weighted.

Table 3 Potential causal association of thyroid function with hepatic fibrosis/cirrhosis

| Outcomes | Exposures | Methods | OR (95% CI) | *P* |
| --- | --- | --- | --- | --- |
| Hepatic fibrosis/cirrhosis | Hyperthyroidism | MR Egger | 0.969 (0.468-2.005) | 0.9342 |
|  |  | Weighted median | 1.166 (0.900-1.513) | 0.2453 |
|  |  | IVW | 1.047 (0.892-1.230) | 0.5748 |
|  | **Hypothyroidism** | MR Egger | 1.390 (0.959-2.015) | 0.0872 |
|  |  | **Weighted median** | **1.430 (1.123-1.821)** | **0.0038** |
|  |  | **IVW** | **1.247 (1.087-1.431)** | **0.0016** |
|  | TSH | MR Egger | 1.648 (0.591-4.591) | 0.4401 |
|  |  | Weighted median | 1.155 (0.875-1.524) | 0.3084 |
|  |  | IVW | 1.146 (0.910-1.444) | 0.2469 |
| CHI3L1 levels | Hyperthyroidism | MR Egger | 1.299 (0.981-1.719) | 0.1012 |
|  |  | Weighted median | 1.041 (0.886-1.222) | 0.6284 |
|  |  | IVW | 1.054 (0.937-1.184) | 0.3813 |
|  | Hypothyroidism | MR Egger | 1.097 (0.837-1.438) | 0.5045 |
|  |  | Weighted median | 0.953 (0.800-1.135) | 0.5874 |
|  |  | IVW | 1.004 (0.898-1.124) | 0.9407 |
|  | TSH | MR Egger | 0.454 (0.176-1.170) | 0.2437 |
|  |  | Weighted median | 0.929 (0.721-1.197) | 0.5680 |
|  |  | IVW | 1.043 (0.865-1.258) | 0.6582 |

OR: odds ratio, CI: confidence interval, MR: Mendelian randomization, IVW: inverse variance weighted, TSH: thyroid stimulating hormone.

Table 4 The causal relationship of thyroid function with hepatic fibrosis/cirrhosis before and after eliminating outliers with heterogeneity.

| Outcome | Exposures | MR analyses | Global test | OR (95% CI) | *P* |
| --- | --- | --- | --- | --- | --- |
| Hepatic fibrosis/cirrhosis | Hyperthyroidism | Raw data | <0.001 | 1.059 (0.803-1.396) | 0.6927 |
|  | Hyperthyroidism | Outlier-eliminated | | 1.129 (0.888-1.434) | 0.3459 |
|  | Hypothyroidism | Raw data | 0.004 | **1.228 (1.042-1.448)** | **0.0171** |
|  | Hypothyroidism | Outlier-eliminated | | **1.266 (1.082-1.482)** | **0.0046** |

MR: Mendelian randomization, OR: odds ratio, CI: confidence interval.
