# Supplementary figures and images for: Thyroid function and hepatic fibrosis/cirrhosis: a two-sample Mendelian randomization study
Source: Front Genet. 2025 Apr 2;16:1399353. doi: 10.3389/fgene.2025.1399353 (PMC11999943; doi:10.3389/fgene.2025.1399353)

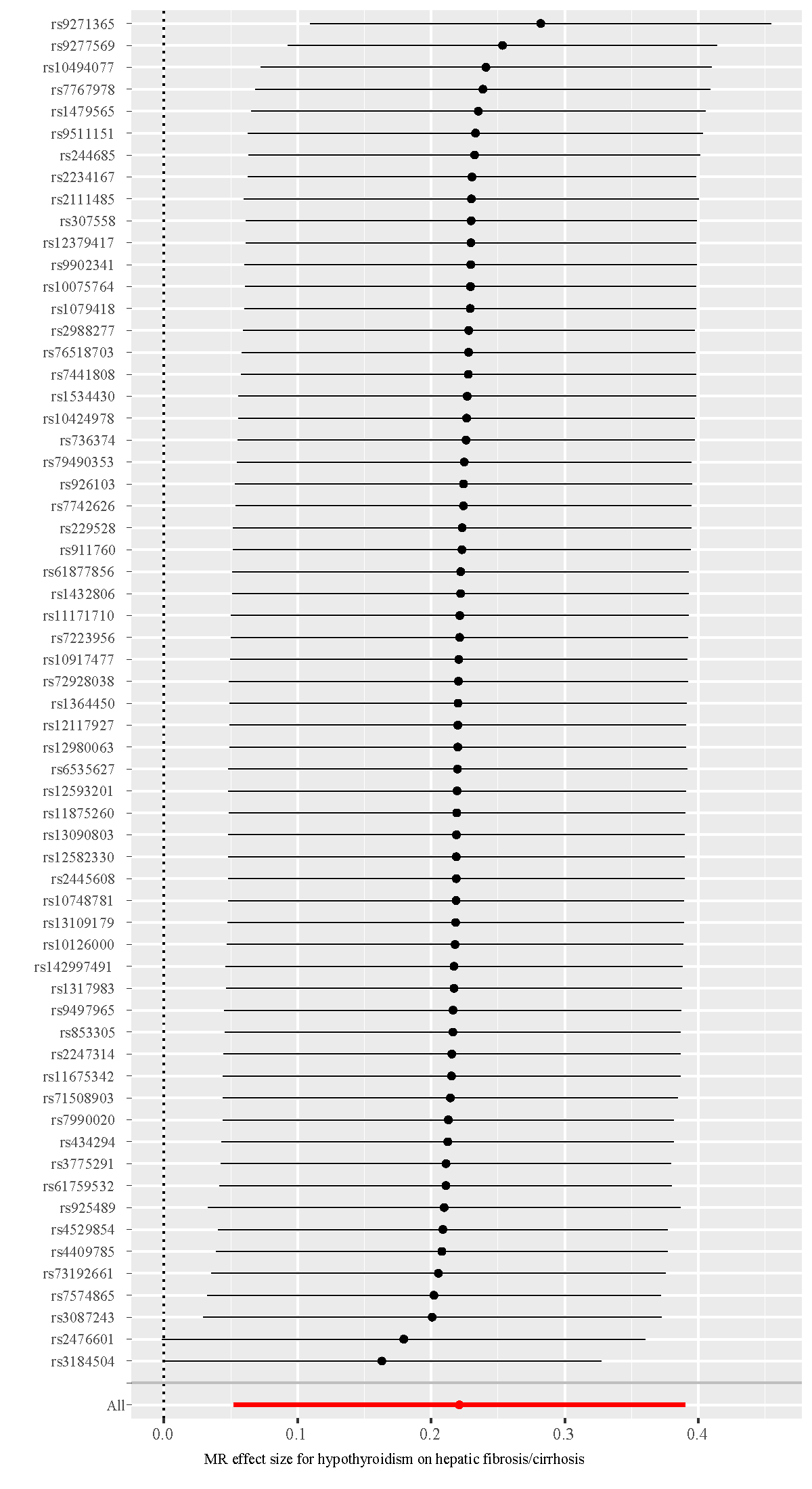

Supplement: Supplementary file 2 [file DataSheet1.ZIP › Raw Data 原始数据和代码-甲状腺功能与肝纤维化肝硬化/Figuers and Tables/Figure 2.tiff]
